# Disruption of Dopamine Homeostasis by Psychostimulants

**DOI:** 10.1007/s12035-026-05769-0

**Published:** 2026-03-05

**Authors:** Juan Torres, Colton Bredenkamp, Atzin Vazquez, Ernest T. Chivero

**Affiliations:** Department of Psychology, University of Nebraska Omaha, Omaha, NE 68182-0001 USA

**Keywords:** Dopamine, Psychostimulant, Cocaine, Methamphetamine

## Abstract

Dopamine is a catecholamine that acts as a modulatory neurotransmitter in the central nervous system. Although dopamine levels are kept within appropriate ranges through various mechanisms, they can be disrupted by multiple factors, including external psychostimulants such as cocaine and methamphetamine. Disruption contributes to cognitive deficits and emotional dysregulation that is associated with psychostimulant-use disorders (PSUD). These stimulants interfere with the dopamine system through various mechanisms that affect its synthesis, storage, release, reuptake, and degradation. Such changes have neurobiological effects, including receptor desensitization, oxidative stress, and neuronal damage, highlighting the importance of maintaining a balanced dopamine system. This review offers a comprehensive overview of how psychostimulants like cocaine and methamphetamine disrupt dopamine synthesis, storage, release, and reuptake, examines the neurobiological consequences of these disruptions, and proposes directions for future research on mechanisms and the development of novel neuroprotective therapies.

## Introduction

Dopamine is a neurotransmitter that controls emotions, motor functions, and feelings of reward and motivation. Maintaining appropriate dopamine levels, through a process known as homeostasis, involves the careful regulation of dopamine production, release, signaling, reuptake, and its inactivation or storage in vesicles [1–5]. Consequently, alterations in these homeostatic processes can significantly impact health, contributing to diseases such as Parkinson’s disease (PD), attention deficit hyperactivity disorder (ADHD), and substance abuse [4–6]. Most dopaminergic neurons originate from the ventral tegmental area (VTA) and substantia nigra (SN). The VTA projects its neurons to the prefrontal cortex (PFC), forming the mesocortical pathway, while projections to the nucleus accumbens create the mesolimbic pathway. Neurons from the substantia nigra project to the dorsal striatum, forming the nigrostriatal pathway. These pathways—the mesolimbic, mesocortical, and nigrostriatal—are linked to reward, emotional control, and motor functions, respectively [4, 7–11].

Psychostimulants, as the name suggests, have stimulating effects on the user and are often used to refer to various drugs, including cocaine and methamphetamine (meth). Cocaine occurs in low concentrations in the leaves of the coca plant, which is native to South America [12–14, 14, 15]. Indigenous peoples of South America traditionally used coca leaves for rituals, medicine, and to reduce hunger and fatigue during long workdays. After the successful extraction of cocaine from these leaves, its use as an anesthetic was discovered. As its popularity grew, cocaine was added to drinks and chocolates and was even used to treat morphine addiction [13, 15]. Feelings of excitement, euphoria, and intense cravings generally occur within minutes of use. However, as the purity and concentration of cocaine increased, its adverse effects became more apparent [12–15]. The rise of freebase and crack cocaine, which can be smoked and are more addictive due to their rapid entry into the central nervous system, further increases the risks associated with cocaine [13, 15]. In the early 1930 s, the popularity of amphetamines led to a decline in cocaine use, but it remains a serious concern today. For example, in the early 2000 s in the USA, cocaine was a leading cause of drug-related visits to the emergency department [13].

In the USA, amphetamines were available without a prescription until the mid-1900s. These drugs were used for weight loss, increasing alertness, and are still used today to treat attention deficit hyperactivity disorder (ADHD) [12, 14]. The amphetamine derivative, methamphetamine, became popular among drug users because it produces feelings of euphoria and intense cravings, despite repeated abuse leading to adverse psychological and physical effects. It is an artificially synthesized drug produced in secret laboratories, mostly found on the West Coast of the USA. Due to the high potential for abuse, the US government increased regulations on its production. However, labs in South American and Southeast Asian countries began to play larger roles in supplying the drug to the USA. As a result, the availability and quality of methamphetamine increased while the price dropped, encouraging widespread use and popularity among drug users [12, 14]. Areas with greater access to methamphetamine, such as the West Coast and states bordering Mexico, have higher rates of individuals seeking treatment for methamphetamine abuse [14]. Although cocaine causes more overdose deaths and emergency department visits, more people use methamphetamine because it is more widely available than other psychostimulants.

Today, psychostimulant use has increased significantly since the early 1900 s, raising concerns about health risks [12–14]. In a previous analysis of over 1 million urine drug tests by Twillman and colleagues, positive tests for cocaine rose nearly 21% from 2013 to 2019, while methamphetamine use increased substantially from 1.43% to 8.29% during the same period [16]. More recently, the 2021 National Survey on Drug Use and Health (NSDUH) showed that among respondents aged 12 and older, 6.0% reported lifetime methamphetamine use, and 0.9% reported using it in the past month. Similarly, 14.6% had used cocaine during their lifetime, with only 1.7% using it in the past year (Substance Abuse and Mental Health Services Administration [SAMHSA] [17]. Overdose deaths involving psychostimulants in the USA have skyrocketed from around 5000 in the early 2000 s to nearly 60,000 in 2022 [18].

### Scope and Structure

Given the widespread use of psychostimulants such as methamphetamine and cocaine, and the critical role of the dopaminergic system, this review aims to provide a comprehensive analysis of how these substances disrupt dopamine homeostasis and their neurobiological effects. The discussion will detail the processes involved in dopamine synthesis, storage, release, reuptake, and breakdown, highlighting how psychostimulants interfere with these key functions. Following this overview, the review will explore the neurobiological consequences of disrupted dopamine signaling, including receptor desensitization, neuronal damage, and tolerance. It will also examine the potential impacts on cognitive function and emotional regulation, as well as interactions with other neurotransmitter systems. The conclusion emphasizes the importance of further research to better understand the mechanisms behind psychostimulant effects and to develop innovative neuroprotective strategies to mitigate adverse outcomes. This structured analysis aims to deepen our understanding of the connection between psychostimulants and dopamine imbalance, ultimately guiding future therapeutic approaches.

## Dopamine System and Its Homeostatic Regulation

Dopaminergic neurons can produce dopamine from its precursor, tyrosine, an amino acid. The synthesis of dopamine relies on the enzyme tyrosine hydroxylase (TH) and is the rate-limiting step in the process [2–4, 6, 19]. When stimulated, dopamine is released into the synaptic cleft, where it activates post-synaptic receptors. Dopamine targets include D1-type (D1 and D5) and D2-type (D2, D3, D4) receptors. Presynaptic D2-type receptors can regulate dopamine release by acting as autoreceptors to reduce dopamine synthesis [4, 19–24]. Excess dopamine can be broken down by catechol-O-methyltransferase (COMT), which exists in two isoforms. The membrane-bound isoform is more common in the brain, while the soluble COMT isoform is found predominantly in tissues [25]. Dopamine can also be degraded by monoamine oxidase B (MAO-B), which is mainly present in human astrocytes. Transporter proteins take dopamine back into the presynaptic neurons’ cytosol for repackaging into vesicles with the help of vesicular monoamine transporter 2 (VMAT2) [2–4, 6, 26–31]. This section discusses and summarizes the key steps in dopamine metabolism and its regulation.

### Dopamine Synthesis

Dopamine is mainly produced in dopaminergic neurons in the substantia nigra and ventral tegmental area [4]. To begin synthesis, phenylalanine is hydroxylated by phenylalanine hydroxylase to form tyrosine. Tyrosine is then converted to dihydroxyphenylalanine (DOPA) by tyrosine hydroxylase. Subsequently, aromatic l-amino acid decarboxylase transforms L-DOPA into dopamine by removing a carboxyl group. The step where tyrosine is converted to L-DOPA is the most critical, since the activity of tyrosine hydroxylase is rate-limiting. In other words, the rate of dopamine synthesis depends on how quickly tyrosine hydroxylase can convert tyrosine into L-DOPA [22, 32–36].

### Storage and Release

The storage of dopamine involves dopaminergic neurons located in the ventral tegmental area and the substantia nigra pars compacta [9, 37]. Dopamine is then packed into vesicles through the activity of VMAT2, which is found in dopaminergic neurons. This process protects dopamine from oxidation, allowing it to be rereleased when needed [6, 27, 28, 38]. If dopamine cannot be packaged into presynaptic vesicles, it becomes vulnerable to oxidation into hydrogen peroxide, superoxide anions, and highly reactive hydroxyl radicals. When enough of these reactive species accumulate, mitochondrial stress increases, which can trigger immune responses and induce apoptosis [6]. Work by Fon and colleagues (1997) implies that VMAT2 is one of the main proteins responsible for recycling dopamine. To this effect, genetic ablation of VMAT2 resulted in significant decreases in dopamine release. This led to issues with feeding, lethargy, and death, all of which are likely due to the depletion of intracellular dopaminergic stores [28]. Literature also indicates that meth disrupts VMAT2 activity, whereas cocaine does not appear to have a direct effect [3, 13].

### Reuptake

Dopamine transporters (DATs) and VMAT2 are crucial for maintaining dopamine balance. The reuptake process begins after a dopaminergic neuron releases dopamine into the synapse. Typically, DATs help transport extracellular dopamine from the synapse back into the presynaptic neuron. VMAT2 then facilitates the transport and storage of dopamine into synaptic vesicles [6, 27, 38]. Interestingly, Jones et al. (1998) demonstrated that blocking other catecholamine transporters or enzymes that break down dopamine does not affect the rate at which dopamine is cleared from the synapse [3]. Only inhibiting DATs significantly prolongs the presence of dopamine in the synaptic cleft. When this occurs, other proteins do not seem to compensate for high extracellular dopamine levels, indicating that DATs are the primary proteins responsible for dopamine reuptake [3]. Moreover, because dopamine must reach the cytosol to be repackaged by VMAT2, dysfunction in DATs can lead to reduced dopamine stores. In fact, genetic inhibition of DATs in mice results in decreased intracellular dopamine levels and lower tyrosine hydroxylase protein expression in the striatum. In addition, tyrosine hydroxylase activity is nearly twice as high in DAT knock-out mice compared to wild-type mice. This suggests that DAT activity is not only essential for reuptake but also plays key roles in regulating synthesis, recycling, and subsequent release [3].

### Degradation

After dopamine is released into the synaptic cleft and interacts with post-synaptic dopamine receptors, signaling ends either through removal or degradation. Removal involves DAT, while degradation involves a series of enzymatic reactions [39]. Enzymes such as MAO, COMT, alcohol dehydrogenase (ADH), aldehyde dehydrogenase (ALDH), and aldose reductase (AR) are known to degrade dopamine [29]. MAO and COMT are the main enzymes responsible for breaking down dopamine into its byproducts [29].

MAO is an oxidoreductase that facilitates the breakdown of catecholamines, including dopamine. MAO exists as two isoenzymes, MAO-A and MAO-B [26]. Both isoenzymes are located in the outer membrane of the mitochondria. However, MAO-A is mainly found in nigrostriatal dopaminergic axon terminals, whereas MAO-B is primarily present in astrocytes and serotonergic neurons [2, 4, 26–31]. It was once thought that MAO-A and MAO-B had similar roles in the dopaminergic system. However, earlier research indicated that MAO-B is responsible for oxidizing most dopamine in humans, while MAO-A is more prominent in mice [31]. Recent evidence, however, challenges this view, suggesting that striatal dopamine is mainly degraded by MAO-A, with MAO-B playing a more significant role in regulating levels of gamma amino-butyric acid [26]. Future research could focus on better understanding how these psychostimulants affect MAO isoenzymes that are involved in dopaminergic degradation. Methamphetamine and cocaine likely interfere with MAO activity, leading to an increase in extracellular dopamine that is prone to oxidation, resulting in oxidative products.

To break down dopamine, COMT converts it into 3-methoxytyramine and the dopamine metabolite 3,4-dihydroxyphenylacetic acid (DOPAC) to homovanillic acid (HVA) [40]. COMT has two isoforms—a soluble cytoplasmic form that is present in the peripheral nervous system and glial cells and a rough ER-bound isoform that primarily exists in neurons [29]. COMT is highly active in glial cells, but there is no clear evidence of activity in nigrostriatal dopaminergic neurons [30]. Further research is needed to understand the effects of psychostimulants on COMT activity in the degradation of dopamine and other catecholamines.

### Regulation of Dopamine Levels

Maintaining appropriate levels of dopamine involves input from multiple sources, including GABAergic, glutamatergic, and dopaminergic signaling mediators. Inputs from glutamatergic and GABAergic interneurons also affect dopaminergic homeostasis. For instance, N-methyl-D-aspartate (NMDA) antagonism can indirectly influence tyrosine hydroxylase activity [22, 41]. When NMDA receptor activity is blocked, Extracellular signal-regulated kinase (ERK) activity does not increase. This is important as ERKs enhance tyrosine hydroxylase activity through phosphorylation after chronic morphine or cocaine use (Berhow et al., 1996). Increased D2 autoreceptor activity is associated with lower extracellular glutamate levels and reduced glutamate release in regions like the nucleus accumbens. By reducing dopamine signaling and glutamatergic activity, heightened D2 autoreceptor activity diminishes communication between the nucleus accumbens and regions such as the VTA and prefrontal cortex [42].

Antagonizing glutamate and GABA receptors in the substantia nigra pars compacta (SNc) increases dopamine levels. In the presence of GABA-A and B receptor antagonists, blocking AMPA receptors has no effect, whereas inhibiting NMDA receptors maintains high dopamine levels, indicating that NMDA and α-amino-3-hydroxy-5-methyl-4-isoxazolepropanoic acid (AMPA) receptors may be involved in different regulatory mechanisms within the SNc even though both are glutamatergic receptors [43]. In rats with Parkinsonian-like symptoms, NMDA administration produces a smaller response from VTA dopamine neurons than in control rats. In addition, inhibiting dopamine D2 and serotonin 2 A receptors helps normalize the abnormal firing of VTA neurons in the same rats. Repeatedly inhibiting D2 receptor activity with risperidone appears to restore serotonin 2A’s inhibitory effect on dopamine release [44]. These mechanisms depend on GABA’s inhibitory action. Previous studies have shown that GABA-A receptors regulate the activity of mesolimbic dopamine neurons; without this regulation, dopamine levels increase when these receptors are blocked [45]. In the dorsolateral and the ventral striatum, both GABA-A and GABA-B receptors regulate dopamine release [46]. Although both receptor types help regulate dopamine activity, they do so in different ways. For example, enhancing GABA-B receptor activity in the VTA has been shown to blunt dopaminergic activity in the NAc in response to stress. Meanwhile, administering GABA-A receptor agonists in the VTA potentiates the stress-induced dopaminergic response in the NAc [47].

From a dopaminergic perspective, D2-dopamine receptors play a crucial role in inhibiting dopaminergic activity. These receptors are located around the dendrites and cell bodies of dopaminergic neurons in the midbrain, as well as in the axon terminals of striatal dopaminergic neurons. Activation of D2 receptors can affect voltage-gated calcium channels, inward-rectifying potassium channels, and other neurotransmitters [42, 48, 49]. When D2 receptors are activated, they trigger signaling pathways that involve PKCβ and phosphorylated ERK, promoting DAT trafficking to the neuronal membrane. This increases the rate at which dopamine is cleared from the synapse, demonstrating how synaptic dopamine levels can be regulated through proteins associated with D2 mechanisms [50]. In addition to lowering dopamine levels, D2 receptor activation also decreases calcium influx in striatal slices of mouse brains. Inhibition of these L-type calcium channels effectively prevents changes in calcium and dopamine levels, suggesting regulation of dopamine release via facilitation of voltage-gated calcium channels [49].

Activity of D2 auto receptors can be disrupted by psychostimulants, such as amphetamines, which separate the dopamine receptor from the inhibitory G-protein it is coupled to [48]. Overstimulation by stimulants can lead to desensitization, resulting in a decrease in signaling activity due to persistent receptor activation [51, 52]. A previous study demonstrated that in mice with diet-induced obesity, the inhibition of VTA D2 autoreceptors was less pronounced than in lean mice, indicating that desensitization of D2 autoreceptors can be influenced by external factors such as obesity [51]. Desensitized D2 autoreceptors were shown to be resistant to trafficking, thereby regulating the localization of these receptors [52].

## Mechanisms of Psychostimulant Action

Both cocaine and methamphetamine play important roles in PSUDs because they impact the dopaminergic system in closely related ways. At the cell signaling level, acute exposure to methamphetamine or cocaine results in extraordinarily high levels of dopamine, increased dopaminergic signaling, and dopamine depletion, which is often described as feeling “washed out” [12, 13].

At the epigenetic level, chronic methamphetamine and cocaine use are associated with epigenetic changes that induce longer-lasting changes in the synthesis, reuptake, storage, and degradation of dopamine, as well as changes in the sensitivity of receptors within the dopaminergic system [5, 6, 22–24, 27, 32, 38, 53, 54].

At the systems level, both cocaine and methamphetamine impact the nigrostriatal, mesocortical, and mesolimbic pathways, although the underlying mechanisms might differ. Disruptions in these areas can lead to fatigue, irritability, and depressed mood. The mesolimbic dopamine system is essential since it is considered the brain’s reward pathway. Disruptions to the ventral tegmental area and nucleus accumbens can cause intense cravings for both drugs, making it more difficult to overcome addiction [4, 8, 12, 13].

Injecting or smoking psychostimulants produces quicker and often stronger effects on the central nervous system compared to snorting. Snorting cocaine or methamphetamine results in slower effects due to bioavailability and the time it takes for the drug to be absorbed into the nervous system [12–15].

## Effect of Psychostimulants on Dopamine Synthesis

### Causality Caveat

Before exploring the upcoming subsections that may include imaging studies, it is important to consider potential causality caveats, especially when interpreting findings from human imaging research. While these studies provide valuable insights into brain structure and function, they often lack causality and are mainly associative. Causality is established when a clear cause-and-effect relationship is demonstrated. An associative relationship only indicates that two or more variables are related [55]. The difference is crucial when explaining the results of a study. Additionally, confounding variables such as environmental factors and individual genetic differences can influence outcomes and make it harder to determine causality. Therefore, while imaging studies can reveal associations between neural mechanisms and behavior, caution is needed when drawing conclusions about cause and effect. Other research methods, like longitudinal studies, often help clarify these relationships.

### Immediate Increase in Dopamine Levels

#### Cocaine

Several studies have shown that cocaine causes a rapid increase in dopamine release by inhibiting the reuptake activity of DATs, leading to higher concentrations of dopamine in the synaptic cleft [56–61]. Cocaine binds to DATs, preventing dopamine from reaching its binding site and stopping the transporter from changing conformation, which results in dopamine accumulating in the synaptic cleft and overactivating the system [57, 60, 62–65]. Other than preventing reuptake, Venton et al. (2006) demonstrated that cocaine also enhanced dopamine release by mobilizing a synapsin-dependent reserve pool of dopamine-containing synaptic vesicles, suggesting that cocaine can boost the exocytotic release of dopamine. Prior to the work of Venton and colleagues (2006), previous studies have shown that there is a readily releasable pool of vesicles that are available for immediate exocytosis and a reserve pool that is spatially segregated and mobilized after prolonged synaptic activity [66–70]. Cocaine can thus mobilize these vesicles during extended periods of prolonged synaptic activity.

In another study, 2 days after a regimen of repeated bi-daily 15 mg/kg cocaine treatments, over 5 days, withdrawing rats had decreased TH-immunoreactive varicosities in the nucleus accumbens core, while these increased in the shell at 14 days post-withdrawal, suggesting the involvement of these changes in behavioral sensitization [36]. However, Licata and Pierce (2004) found that tyrosine hydroxylase activity is not affected by a single dose of 15 mg/kg cocaine or by repeated treatment for a week, suggesting that dopamine synthesis is not altered by cocaine administration. Thus, psychostimulants appear to alter the regulation of already existing dopamine stores [35].

#### Methamphetamine

Similar to cocaine, studies with methamphetamine show an immediate increase in dopamine. In one study, rats given a binge dose of four injections of 4 mg/kg methamphetamine 2 h apart experienced increased dopamine levels in the frontal cortex for up to 24 h, but striatal levels remained unchanged [71]. Furthermore, significant increases in tyrosine hydroxylase-positive axons were observed in the frontal cortex shortly after meth treatment [71].

### Downregulation of Dopamine Receptors

Following long-term use of psychostimulants, the brain attempts to compensate for abnormal dopamine levels by upregulating or downregulating the number of dopamine receptors, resulting in changes in sensitivity [5, 72, 73]. Downregulation of receptor numbers leads to decreased sensitivity to dopamine, resulting in tolerance and, therefore, drug intake escalation.

#### Cocaine

Neuroimaging studies have shown that there are fewer dopamine D2 and D3 receptors available in the brains of cocaine users compared to individuals who do not use cocaine [74, 75]. The decrease in dopamine D2 receptors has been observed in regions such as the putamen, striatum, and caudate [54]. Prolonged exposure to cocaine has also resulted in requiring more dopamine to activate D2 receptors in the nucleus accumbens [76], suggesting changes in receptor sensitivity. One possible explanation for this change is that cocaine alters the number of D2 receptors available, or the expression and coupling of the relevant G-protein subunits that facilitate signaling of these metabotropic receptors. Gong and colleagues (2021) demonstrated that changes in the expression of Ga_o_ subunits in D2 receptors are a more likely cause. Cocaine-induced reductions in D2 sensitivity were potentiated when Ga_o_ expression was experimentally decreased but were prevented when Ga_o_ was overexpressed [76]. The sensitivity of D2 receptors returned to baseline after approximately a week of cocaine abstinence, affecting only the nucleus accumbens, since D2 receptor sensitivity and Ga_o_ subunits were not affected in other regions [76]. However, levels of Ga_o_ proteins have also been observed to decrease in the ventral tegmental area while cAMP-mediated signaling increases in the nucleus accumbens. These changes in G-protein alpha subunits suggest a shift in the balance between dopamine receptor types, resulting in reduced inhibition in the VTA and leading to increased excitatory activity in the nucleus accumbens [72].

Park et al. tested the hypothesis that chronic cocaine use unbalances D1 and D2 receptor-mediated signaling during cocaine intoxication by using microprobe optical imaging to compare dynamic changes in intracellular calcium [77]. Results from this study demonstrate that chronic cocaine use drastically reduces cocaine-induced dopamine signaling, shifting the balance between D1R and D2R signaling during intoxication toward favoring D1R (stimulatory) over D2R (inhibitory) signaling, leading the authors to predict that this shift may facilitate compulsive intake in addiction [77]. In this context, another study showed that the levels of D2R expression remain unchanged in the nucleus accumbens of tree shrews withdrawing from cocaine; however, D1 receptor expression increases, accompanied by the presence of voltage-gated calcium channel 1.2 (Cav 1.2) [78]. However, antagonizing D1 receptors decreases the expression of Cav 1.2 and cocaine-seeking behaviors after a prolonged period of cocaine withdrawal [78]. These increases in D1R activity and subsequent cAMP-mediated signaling pathways are commonly observed during withdrawal and likely contribute to the relapse of cocaine users [72].

Deep brain stimulation (DBS) and DBS-like approaches have been used to examine the effects of stimulating neurons containing D1 and D2 receptors in the treatment of resistant substance use disorders. For example, using optogenetic deep brain stimulation to target D2 receptors in the nucleus accumbens attenuates cocaine seeking in male rats, but not in female rats. Meanwhile, stimulating D1 receptors does not affect cocaine seeking regardless of sex [79].

In a more recent study, high-frequency stimulation of the nucleus accumbens resulted in lower tonic dopamine levels compared to control rats. It prevented an increase in dopamine levels following cocaine treatment, resulting in dopamine levels similar to those of the control group [80]. This enhanced dopamine clearance may be due to the activation of more D1 and D2 receptors, compared to normal conditions, or the activation of receptors with a lower affinity for dopamine [81]. Rahman and McBride (2001) suggest that simultaneous activation of D1 and D2 receptors is necessary for dopaminergic homeostasis. For instance, significant reductions in extracellular dopamine levels occurred in the nucleus accumbens when both receptor types were activated. Activating each receptor individually caused smaller changes [81].

#### Methamphetamine

In a human PET study, Volkow et al. found that low levels of brain dopamine D2 receptors in methamphetamine abusers were associated with low levels of glucose metabolism in the orbitofrontal cortex [82]. Low levels of D2 receptor availability have also been reported in cocaine, alcohol, and heroin abusers [75, 83, 84]. Individuals with more severe methamphetamine addictions have reported more difficulties in regulating emotions, which were also related to lower numbers of D2-type dopamine receptors in the amygdala, compared to healthy controls [85]. While these studies offer insights into what may be occurring to D2-type dopamine receptors after methamphetamine use, longitudinal studies suggest that changes to D2 dopamine receptor availability are a consequence of various factors. In aging mice, striatal D2 dopamine receptor availability decreased over time [86]. Older adults observed over a 5-year period have also been shown to experience age-related decreases in D2 receptor availability due to aging [87]. Even in younger study subjects, levels of D2 receptors appear to decrease as their dopamine system matures [88]. Without ruling out alternative explanations, it is difficult to establish a clear cause-and-effect relationship of altered D2 dopamine receptor levels [82, 85].

High doses of methamphetamine can activate D1 dopamine receptors, leading to increased calcium concentrations and cAMP levels [73]. Following these increases in second messengers, protein kinase A (PKA) becomes active and subsequently activates cAMP response element binding protein (CREB). When CREB is phosphorylated, it induces transcription and alters the expression of tyrosine hydroxylase, the rate-limiting enzyme for dopamine synthesis [5, 73]. Methamphetamine-induced activation of D1 receptors results in effects similar to addiction, such as increased conditioned place preference scores and increased c-Fos expression in neurons of the orbitofrontal-dorsal striatum pathway of mice [89]. Blocking D1-like receptors effectively prevents these effects; however, caution is needed to distinguish between D1 and D5 dopamine receptors in such experiments, as D5 receptors play a crucial role in regulating methamphetamine-induced changes to DAT and locomotor activity [89, 90].

### Long-Term Changes in Tyrosine Hydroxylase

#### Cocaine

The expression of tyrosine hydroxylase in healthy controls and cocaine-dependent individuals is comparable [91]. However, cocaine dependence is associated with increased methylation of the tyrosine hydroxylase gene body in the nucleus accumbens in humans, which is associated with cocaine-seeking and chronic cocaine exposure [91]. In another study, chronic cocaine administration was shown to reduce tyrosine hydroxylase mRNA levels in the substantia nigra pars compacta (SNc) 1 day after cocaine treatment in mice [92]. In addition, regional differences in tyrosine hydroxylase expression exist in areas such as the VTA, which is particularly sensitive to cocaine-induced changes in tyrosine hydroxylase [22, 41]. In rats chronically treated with 10 mg/kg cocaine, tyrosine hydroxylase activity becomes elevated in the VTA 1 week after the final cocaine dose and remains elevated for up to 12 weeks [22]. In another chronic treatment model, treatment with cocaine or morphine increased the activity of extracellular signal-related kinase in the VTA, which is linked to the subsequent increases in tyrosine hydroxylase expression. Interestingly, antagonism of NMDA and D2 receptor activity prevents changes in tyrosine hydroxylase activity, whereas antagonism of the D1 receptor had no effect [22, 41].

In another study, Trulson et al. demonstrated that chronic cocaine administration (10 mg/kg, IP, every 12 h for 10 consecutive days) depletes tyrosine hydroxylase immunoreactivity in the mesolimbic dopamine system in the rat brain [93].

#### Methamphetamine

Previous studies have shown that long-term treatment of rats with methamphetamine (20 mg/kg, IP, every 12 h for 10 days) causes a significant reduction in tyrosine hydroxylase-stained axons and terminal boutons in the nucleus accumbens, frontal cortex, ventral tegmental area, and caudate nucleus regions [93, 94]. Tissues from these studies were examined 60 days after the drug regimen ended. This data indicates that chronic methamphetamine administration leads to a long-lasting loss of tyrosine hydroxylase enzyme in the striatum and other limbic structures.

In a 10-day methamphetamine self-administration rat model, levels of tyrosine hydroxylase mRNA and protein significantly increased in both the VTA and SNc 1 day after forced abstinence. By day 30, tyrosine hydroxylase mRNA and protein levels had returned to normal [95].

Repeated methamphetamine treatments of 2.5 and 10 mg/kg over a week cause decreases in tyrosine hydroxylase mRNA and protein levels in the substantia nigra and striatum of both wild-type and mu-opioid receptor knock-out mice [96]. Immunostaining shows that methamphetamine reduces the number of tyrosine hydroxylase-positive neurons in the substantia nigra of wild-type mice but not in mu-opioid receptor knock-out mice [96]. Therefore, mu-opioid receptors may serve as important pharmacological targets because they are involved in the mechanisms underlying meth-induced loss of dopaminergic axons and neurons in the nigrostriatal pathway [96]. Similarly, wild-type rats self-administering methamphetamine over 10 days also experience decreases in tyrosine hydroxylase proteins and mRNA in the substantia nigra and ventral tegmental area [95]. In one study, wild-type mice treated acutely with four injections of 8 mg/kg methamphetamine given every 2 h showed significant reductions in tyrosine hydroxylase expression, phosphorylation, and activity in the striatum and substantia nigra at 3 days after injection. Additionally, dopamine levels in the striatum were significantly reduced. However, these effects could be prevented with pretreatment using liposomal melatonin or by genetically inhibiting protein kinase Cδ (PKCδ), indicating that PKCδ plays a role in modulating tyrosine hydroxylase activity [97].

In binge dosing models using adult male mice, four injections of meth, ranging from 4 to 8 mg/kg, reduce levels of dopamine, dopamine metabolites, and tyrosine hydroxylase in the striatum. These reductions are observed 1 day after methamphetamine administration and remain significant for up to a week [98]. Dang et al. showed that meth treatment increases PKCδ and protein phosphatase 2 A (PP2A) activity while decreasing tyrosine hydroxylase phosphorylation at serine 40, dopamine levels, and PKA activity in the mouse striatum [99]. Treatment with rottlerin, a PKCδ inhibitor, attenuated the effects of methamphetamine. Any benefits of PKCδ inhibition were counteracted by treatment with a PKA inhibitor (H89) or a PP2A activator (FT7720) [99]. This data suggests that PKA and PP2A are key proteins in maintaining dopamine homeostasis, while PKCδ indirectly contributes to methamphetamine-induced decreases in dopamine levels and tyrosine hydroxylase phosphorylation by lowering PKA activity and increasing PP2A activity [97–100].

Adolescent mice injected with a single dose of 4 mg/kg of methamphetamine show behavior changes and exhibit more anxiety-like symptoms, yet the total protein and phosphorylation levels of tyrosine hydroxylase in the hippocampus remain unaffected [101]. In a separate study, injecting the ventral tegmental area with an adeno-associated virus (AAV) to produce overactive tyrosine hydroxylase in mice increases their sensitivity to methamphetamine. Overactive tyrosine hydroxylase boosts the activity of tyrosine hydroxylase as expected, but dopamine levels in the striatum do not change. However, the striatum shows increased levels of dopaminergic metabolites [102]. Similarly, genetically engineering the overexpression of tyrosine hydroxylase is associated with higher levels of phosphorylated tyrosine hydroxylase, extracellular dopamine, reactive metabolites, and intermediates such as H_2_O_2_ and 3,4-dihydroxyphenylacetaldehyde (DOPAL) [5]

Taken together, levels of tyrosine hydroxylase and dopamine increase immediately following methamphetamine exposure but tend to decrease afterward [5, 71, 97, 98, 101, 102].

## Effect of Psychostimulants on Dopamine Storage

### Alterations in Vesicular Monoamine Transporter Expression

Chronic psychostimulant use alters the expression of VMAT2, which is essential for the transportation and storage of dopamine. This alteration impacts dopamine regulation and can contribute to the development of neurotransmitter imbalances and addiction.

#### Methamphetamine

Methamphetamine competes with dopamine to bind to VMAT2, which reduces dopamine binding to VMAT2. As a result, less dopamine is packaged into vesicles and instead remains in the cytosol [6, 27, 38, 103]. Exposure to methamphetamine can decrease the number of VMAT2 proteins and the number of presynaptic vesicles available for storing dopamine [27]. These disruptions in dopaminergic repackaging into vesicles are concerning because dopamine remaining in the cytosol is vulnerable to oxidation, which can lead to neurotoxicity [6, 103].

Furthermore, meth-induced changes to VMAT2 vary with age in rodent models. In young adult rats (postnatal day 90), high-dose administration of methamphetamine causes significant reductions in dopamine uptake by VMAT2 after 1 h and 7 days post-treatment compared to adolescent (postnatal day 40) rats, demonstrating dynamic changes in VMAT2 susceptibility [104].

When VMAT2 proteins become dysfunctional, monoamine exocytosis cannot occur. In a study by Fon et al. (1997), mice born homozygous-negative for the VMAT2 gene show minimal movement and poor feeding, ultimately dying within 7 days after birth. In these mice, vesicles are unable to release dopamine or package cytosolic dopamine into vesicles. Instead, dopamine is rapidly metabolized [28]. However, when VMAT2 homozygous-negative mice receive chronic amphetamine treatment, their movement increases and feeding occurs, prolonging their survival. Importantly, Fon and colleagues (1997) demonstrated that amphetamines can alter dopamine levels independently of VMAT2. Instead, amphetamines cause dopamine transporters to efflux cytosolic dopamine and promote dopamine synthesis [28]. A human emission tomography (PET) study showed that early on in abstinence from methamphetamine, more [^11^C]-(+)-dihydrotetrabenzaine (+)[^11^C] (DTBZ) binds to VMAT2. Since dopamine competes with DTBZ to bind at the same site, increased (+)[^11^C]DTBZ binding to VMAT2 suggests less dopamine is present within vesicles. After a week of abstinence from methamphetamine, striatal binding of (+)[^11^C]DTBZ to VMAT2 was similar to that of healthy controls [103].

In addition to the direct effects on VMAT2, methamphetamine also impairs the activity of D1 and D2 receptors [20, 105–107]. These receptors play a role in the cascade of events that load dopamine into vesicles in neurons. When D1 and D2 receptor function decreases, dopamine becomes more concentrated in the cytosol, where it is prone to oxidation and degradation [20]. As mentioned in an earlier section, exposure to methamphetamine is associated with the downregulation of D2 receptors, and as expected, methamphetamine did not have any effect on D1R^−/−^ knock-out mice, as these mice lack the D1 dopamine receptors [105–107].

#### Cocaine

Cocaine is primarily considered a dopamine uptake inhibitor, and unlike methamphetamine, it has not been shown to inhibit VMAT2 [57, 59, 108]. However, cocaine does seem to influence vesicular dopamine [108]. In fact, cocaine can deplete vesicles near the plasma membrane and also reduce reserve pools of dopamine [108]. Neurons in the ventral tegmental area and nucleus accumbens are particularly vulnerable to this effect [59]. These limbic structures experience immediate increases in synaptic dopamine, accumulation of ΔFosB, and subsequent changes in gene and protein activity. Within the mesocorticolimbic pathway, the increase in ΔFosB and the transcriptional changes that follow are key features of cocaine addiction [59].

In rat models, cocaine treatment causes VMAT2 to move away from the synaptic plasma membrane into the intracellular space. Farnsworth and colleagues (2009) observed that D2-dopamine receptors mediate cocaine-induced trafficking of VMAT2. When they blocked D2 receptors with eticlopride, cocaine-induced VMAT2 trafficking did not occur [109].

In a 2006 study, Venton et al. revealed that cocaine depletes intercellular stores of dopamine by mobilizing a synapsin-dependent reserve pool, leading to increased dopamine release [108]. After cocaine treatment, VMAT2 levels have been shown to decrease in the striatum of non-human primates [110]. In this study by Narendran et al. (2015), the researchers minimized confounding variables, ensuring they were confident that VMAT2 reductions were caused by cocaine treatment. In human brain tissue, decreases in striatal VMAT2 are also observed in cocaine abusers. These findings require further validation since polysubstance use that is common in humans may also contribute to decreases in VMAT2, making it difficult to establish causality between cocaine use and subsequent reductions in VMAT2 [111–113].

## Effect of Psychostimulants on Dopamine Reuptake—Alterations in Transporter Protein Expression

### Methamphetamine

Methamphetamine-induced neurotoxicity results from the disruption of transport proteins such as DAT and VMAT2, which cause abnormal extracellular and cytosolic dopamine levels [6]. Methamphetamine impairs DAT’s ability to transport dopamine from the synapse back into the presynaptic neuron, a process known as reuptake. Consequently, extracellular concentrations of dopamine increase [6, 27, 38]. The same increase can occur if DAT reverses its function, transporting dopamine out of the cell, back into the synapse [27]. DATs rely on the movement of Na^+^/Cl^−^ ions, and methamphetamine enhances the flow of these ions in and out of the cell [27].

Mice given binge doses of methamphetamine have significantly lower levels of DATs in their striatum compared to controls, and this effect lasts up to a week [97, 98]. In PKCδ knock-out mice and mice pretreated with liposomal melatonin, meth-induced decreases in DATs are attenuated [97]. PKCδ activity appears to influence dopamine reuptake in addition to dopamine synthesis, and its activity can be suppressed through genetic inhibition or pretreatment with rottlerin or liposomal melatonin [97–100].

Mice with overactive tyrosine hydroxylase are more vulnerable to the effects of high doses of methamphetamine, leading to lower levels of dopamine transporter expression 2 days after treatment [5]. In wild-type mice, three injections of 3 mg/kg methamphetamine are sufficient to reduce dopamine transporter levels in the striatum. The use of 7,8-dihydroxyflavone (7,8 DHF), an agonist for tropomyosin-related kinase, can diminish these effects [114].

Repeated exposure to methamphetamine at both low and high doses reduces [^125^I] RTI-121 binding, indicating fewer available dopamine transporters for binding. At 10 mg/kg of methamphetamine, these reductions are more significant than after repeated administration of 2.5 mg/kg. In the striatum of mu-opioid receptor knock-out mice, meth-induced decreases in DAT levels are greater than those seen in wild-type mice [96].

### Cocaine

Cocaine binds to DATs and impairs their function, resulting in increased extra-synaptic dopamine levels and overstimulation of post-synaptic dopamine receptors [53, 54]. To help remove this excess synaptic dopamine, the number of DATs is upregulated, which is often linked to cocaine users’ feeling a “crash” due to the sudden decrease in synaptic dopamine [54]. DAT mRNA levels increase as soon as 2 h after cocaine treatment but become depressed after about a day. Sensitizing mice with cocaine prior to cocaine challenge exacerbates these changes to DATs, suggesting that repeated cocaine use leads to increased sensitivity in the dopaminergic system [92]. Repeatedly administering a dose of 20 mg/kg of cocaine to male and female rats causes large migrations of striatal intracellular dopamine transporters to the plasma membrane. When a lower dose of 5 mg/kg of cocaine is used, this effect is only observed in females. Interestingly, the intracellular DATs normalize more quickly for females than for males, indicating that females recover faster from DAT migration [62]. Therefore, although females tend to be more susceptible to cocaine use and its effects, they also tend to recover more quickly from cocaine-induced changes to DATs [62].

Inhibiting DAT function has downstream effects in areas such as the frontal cortex [115]. When synaptic dopamine is not cleared due to a lack of DAT activity after acute cocaine exposure, excitatory neurons in the frontal association cortex (FrA) of male mice become inactivated. Depleting dopamine stores from neurons in the ventral tegmental area, but not the substantia nigra, prevents this effect. Using D1 and D2 receptor agonists in the medial prefrontal cortex (mPFC) results in more pronounced inhibition of FrA neurons, whereas antagonists for dopamine receptors and other monoamines have no effect [115]. Collectively, Wang et al. (2023) provide strong evidence that D1-positive and D2-positive excitatory neurons in the mPFC mediate cocaine’s inhibitory effect on the frontal association cortex. This proposed VTA-FrA-mPFC pathway influences cocaine-induced locomotor sensitization and likely underlies cocaine seeking along with other drug-related behaviors [115]. Previous reports have also shown that cocaine inhibits the electrical activity of dopaminergic neurons and that cocaine has differential effects on dopaminergic neuronal firing in awake and anesthetized rodents [116]. In anesthetized rats, these authors show that cocaine (10 mg/kg, i.p) produced a general decrease of the firing rate and bursting of dopamine neurons, but in awake rats, however, injection of cocaine led a decrease in firing rate and bursting in only 14% of dopamine neurons, but most of the other dopaminergic neurons underwent increases in firing rate and bursting that was correlated with locomotor activity in 52% of the neurons [116]

## Effect of Psychostimulants on Dopamine Degradation

### Methamphetamine

 In this section, we discuss how methamphetamine and cocaine affect MAO and COMT enzymes given their importance in dopamine degradation [37, 117–121].

In mouse models, administering methamphetamine significantly raises mitochondrial oxidative stress in dopaminergic neurons of the substantia nigra pars compacta and VTA, mediated by monoamine oxidases. Blocking of MAO reduces mitochondrial stress in this model. Although chronic methamphetamine causes increased mitochondrial stress in dopaminergic neurons of both the SNc and VTA, these regions are affected differently [118]. SNc neurons suffer axonal and cell body degeneration due to mitochondrial stress, while VTA neurons also experience stress but do not undergo degeneration [118]. In both regions, inhibiting MAO-B activity decreases oxidative stress. One probable reason for SNc axon degeneration is the heightened activity of L-type Ca^2+^ channels, which can increase oxidative stress in the cell body. Inhibiting L-type Ca^2+^ channels helps ameliorate meth-induced mitochondrial stress in SNc axons [118]. Though VTA axons also show increased L-type Ca^2+^ activity, it does not elevate mitochondrial stress. The differences suggest that VTA axons are less vulnerable to L-type Ca2+ channel or MAO-dependent mitochondrial stress compared to SNc axons [118]. Additionally, when examining methamphetamine-induced mitochondrial stress in SNc neuron cell bodies, pretreatment with the MAO-B inhibitor, rasagiline, reduced mitochondrial stress. Conversely, treatment with israpindine, a negative allosteric modulator of Cav1 channels, effectively reduced meth-induced mitochondrial stress in SNc neuron somas but did not affect stress in axons [37]. Importantly, either approach—using an MAO-B inhibitor or an allosteric Cav1 channel modulator to decrease mitochondrial stress in axons and somas—is sufficient to prevent SNc dopaminergic neuron degeneration [37]. Overall, MAO contributes to the loss of dopaminergic neurons in the SNc due to MAO-dependent mitochondrial stress caused by chronic methamphetamine, leading to changes in movement since the nigrostriatal pathway is associated with motor control. Processes related to rewards and emotional regulation may be less affected as these are often associated with the VTA [4, 7–10, 37, 118].

The efficiency of COMT in degrading dopamine is influenced by single-nucleotide polymorphisms at the Val158Met gene. Variations at this position result in altered levels of extracellular dopamine, which affect executive function [117, 121]. The Val allele is associated with increased dopamine degradation and lower executive function resulting from reduced dopamine levels. In contrast, the Met allele is associated with slower dopamine degradation and higher executive function. In a study by Cherner et al., individuals with a homozygous Met genotype showed significantly higher composite executive function scores compared to those with a Val carrier genotype [117]. However, among chronic methamphetamine users, Met carriers had significantly lower scores than Val carriers. Saloner et al. found that in methamphetamine users, Met/Met carriers exhibited lower dopamine levels than Val/Val participants, which may explain the lower scores observed by Cherner et al. in methamphetamine users [117, 121]. The relationship between COMT and substance use varies depending on the context. Factors such as gender differences or the specific brain region studied influence the outcomes [122]. 

### Cocaine

In a study by Pepper et al. [120], pretreating male rats with clorgyline, an inhibitor of monoamine oxidase A, prior to cocaine challenge did not significantly alter extracellular dopamine levels. However, it did significantly reduce dopamine metabolites [120]. Thus, by observing lower dopamine metabolites without an increase in dopamine levels, the researchers believe that both MAO-A and MAO-B activity must be altered to change extracellular dopamine levels [120].

Lowering the degradative activity of MAO-B is beneficial in the context of human cocaine users. A 7-day treatment with selegiline (deprenyl), an inhibitor of monoamine oxidase B, significantly reduced the euphoric effects caused by cocaine in human participants with cocaine dependence [123].

Inhibiting MAO-B activity in cocaine-naive mice, with pargyline or deprenyl pretreatment, reduces cocaine self-administration compared to saline or the MAO-A inhibitor, clorgyline [119]. Pretreatment with pargyline, deprenyl, and clorgyline for 24 consecutive days does not alter dopamine levels in areas such as the frontal cortex, striatum, or nucleus accumbens. However, pretreatment with pargyline and deprenyl decreases levels of dopamine metabolites DOPAC and 5-hydroxyindoleacetic acid (5-HIAA) in the frontal cortex. Ho and colleagues noted that these findings may not apply beyond cocaine-naive individuals, since MAO-B inhibition might not reverse long-term changes to the dopaminergic system seen in experienced cocaine users’ frontal cortex, striatum, or nucleus accumbens [119].

**Fig. 1:**
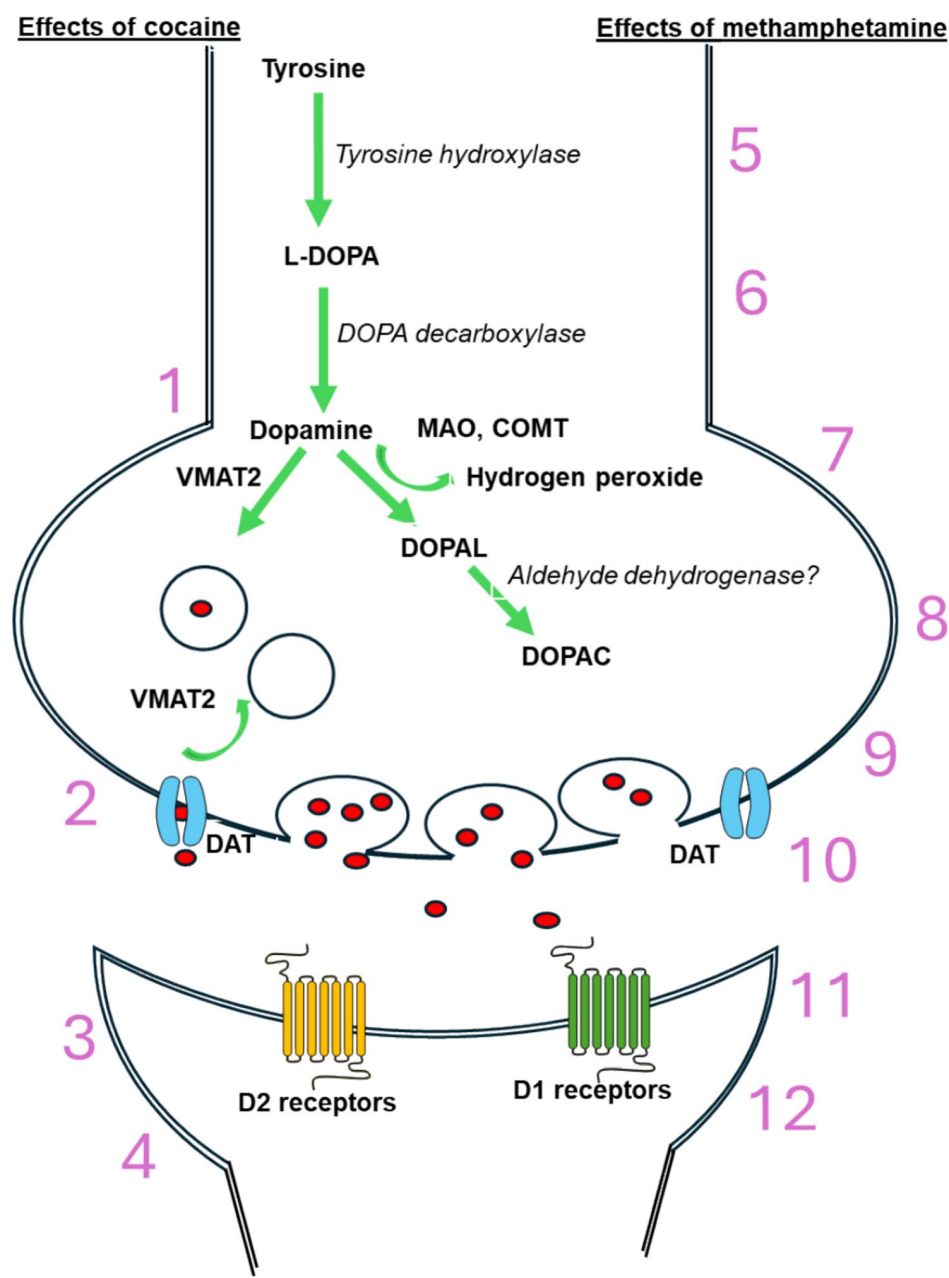
Effects of cocaine and methamphetamine on the dopamine pathway. Dopamine is produced from tyrosine via the action of tyrosine hydroxylase and dopa decarboxylase. Dopamine is then stored in vesicles with the aid of vesicular monoaminergic transporter-2 (VMAT-2). Dopamine can be degraded by monoamine oxidase (MAO) or catechol-O-methyltransferase (COMT) to form homovanillic acid or the metabolites DOPAL and DAPAC and hydrogen peroxide (H_2_O_2_). (1) Dopamine release is decreased in chronic use. (2) Cocaine blocks DAT, increasing synaptic dopamine levels, but does not directly affect VMAT2. (3) D2Rs become downregulated due to chronic drug use. (4) D1Rs become downregulated due to chronic drug use. (5) Increased tyrosine hydroxylase expression. (6) Dopamine release increases after acute use but decreases after chronic use. (7) VMAT-2 function is dysregulated. (8) Methamphetamine competitively binds to DAT and enters intracellular compartment. (9) DATs are downregulated in chronic use. (10) Meth-induced DAT reverse action increases synaptic dopamine levels. (11) D1Rs become upregulated due to chronic drug use. (12) D2Rs become downregulated due to chronic drug use

The rs4680 single-nucleotide polymorphism, often referred to as Val158Met, was carried more often by cocaine dependent individuals, further indicating that the Met and Val alleles play key roles in mediating the effects of stimulants like cocaine and methamphetamine [124]. The overall effects of cocaine and methamphetamine on specific steps of the dopamine pathway are shown in Fig. 1.

## Long-Term Neurobiological Impacts of Psychostimulant-Induced Dysregulation of Dopamine Homeostasis

### Neuroadaptation

#### Receptor Desensitization

Dopamine receptor desensitization occurs after repeated exposure to high dopamine levels. Receptors become less responsive to dopamine, requiring higher drug doses to produce the same effects. In response to desensitization, receptors like D2 are internalized, making signaling more difficult because fewer receptors remain on the plasma membrane [51, 52]. Consequently, D2 receptor function is inhibited, further impairing their autoreceptor activity.

The ventral tegmental area, nucleus accumbens, substantia nigra, and prefrontal cortex are believed to participate in cocaine-induced behavioral sensitization [92]. Common targets include changes to DAT, tyrosine hydroxylase, and D2 receptor expression. Belin and colleagues studied the transcriptional adaptations of neurons in response to cocaine challenge after a long period of abstinence from repeated cocaine exposure [92]. They showed that changes in D2 mRNA levels occur rapidly after cocaine challenge injections. The most pronounced changes in the VTA and substantia nigra pars compacta occurred 24 h after challenge injection for mice pretreated with cocaine, followed by an injection with vehicle. This change was also observed in mice receiving cocaine pretreatment and challenge injection. After 24 h, mice given a cocaine challenge injection showed significantly lower levels of DAT mRNA compared to control mice, regardless of pretreatment, suggesting that cocaine alters the transcriptional response of dopaminergic neurons to a new cocaine challenge even after a long period of abstinence from repeated exposure to the drug [92]. These authors demonstrated that adaptations within the dopaminergic system occur rapidly in response to cocaine exposure, even after a long period of abstinence.

The pathway from the ventral tegmental area to the nucleus accumbens and prefrontal cortex is often a key focus in studies of addiction. In this mesolimbic pathway, stimulant use can cause changes in the morphology of neurons, regulate dopamine receptor expression, and alter the activity of second messengers, tyrosine hydroxylase, and the transmission of non-dopaminergic neurons [10]. In the ventral tegmental area, dopaminergic neurons respond differently to intravenous cocaine administration; some are inhibited, while others are excited [7]. Activity in excitatory neurons can take up to 2 min after drug exposure begins and lasts between 2 and 5 min. Inhibitory activity occurs quickly, within about 20 s after cocaine exposure, and lasts around 5 min. However, peak activity in both inhibitory and excitatory neurons happens approximately 2 min after drug exposure [7]. In a study by Rodriguez-Espinosa and Fernandez-Espejo (2015), repeated cocaine administration led to significantly increased distances traveled by rats compared to saline-treated controls. Additionally, cocaine-treated mice traveled significantly further from day 1 to the 3rd, 8th, and 18th days of the experiment. These differences in locomotor activity between saline and cocaine-treated mice indicate biochemical changes caused by repeated drug use [10]. Indeed, the activity of protein kinase A and tyrosine hydroxylase was significantly higher in the nucleus accumbens of cocaine-treated mice on the third day of the study. On days 8 and 18, tyrosine hydroxylase expression was significantly elevated in the ventral tegmental area [10].

#### Synaptic Plasticity

Another long-term neuroadaptation involves changes in synaptic plasticity, which is the ability of synapses to strengthen or weaken over time. Synaptic plasticity changes can influence learning and memory processes in the hippocampus [125]. Previous studies have demonstrated that structural alterations, including changes in dendritic spine density and morphology, occur [73].

Recently, Santos et al. (2018) used a locomotor sensitization paradigm to assess changes in synaptic plasticity in the striatum caused by chronic cocaine exposure. They found that a higher percentage of axon terminals were closer to their receptor targets in the ventral striatum of mice treated daily with cocaine compared to mice treated with saline. In the nucleus accumbens shell of mice exposed to cocaine, dendritic spine density increased significantly by about 13% compared to control mice [73]. Overall, this data suggests that chronic cocaine use induces changes in synaptic plasticity by altering the spacing between axon terminals and receptor targets in the ventral striatum, as well as increasing dendritic spine density in the NAc shell [73].

### Neurotoxicity

#### Oxidative Stress

The excessive release and accumulation of dopamine in the context of psychostimulant use can lead to the production of reactive oxygen species (ROS), causing oxidative stress. Oxidative stress is known to damage cellular components, including proteins, lipids, and DNA, leading to impaired cell function and cell death. Although oxidative stress caused by cocaine and methamphetamine is well documented [37, 38, 126], much of the evidence is preclinical. There is a paucity of human clinical evidence demonstrating the effects of antioxidant strategies on outcomes in psychostimulant use disorders, highlighting the translational gap between oxidative stress mechanisms and treatment [127, 128].

Decreases in striatal cytoplasmic dopamine, achieved by inhibiting tyrosine hydroxylase with AMPT (alpha-methyl-p-tyrosine), protect against meth-induced damage in dopaminergic neurons. However, increases in cytoplasmic dopamine, whether by inhibiting dopamine storage, dopamine metabolism, or increasing dopamine precursors, lead to oxidative stress and subsequent microglial activation, resulting in neurotoxicity [129].

#### Neuronal Damage and Cell Death

Prolonged oxidative stress and the resulting cellular damage can ultimately cause neuronal apoptosis (programmed cell death), necrosis (unplanned cell death), or pyroptotic cell death [12]. This neuronal loss may contribute to long-term cognitive and behavioral deficits observed in individuals who chronically use psychostimulants.

Using methamphetamine and cocaine alone or together can lead to neuroinflammation and neuronal cell death [5, 12, 16, 126]. These drugs also increase the likelihood of strokes, seizures, heart problems, hyperthermia, and death [6, 12, 38, 126]. Although both cocaine and methamphetamine cause and worsen cardiac complications, cocaine is linked to more overdose deaths related to cardiotoxicity [12]. Moreover, drug users are more likely to contract sexually transmitted diseases and experience psychological disturbances such as psychosis, anxiety, and major depression compared to non-drug users [12–14]. Hyperthermia caused by cocaine and methamphetamine is a significant side effect often seen with overdose. Past studies show notable increases in body temperature due to drug use. In animal models, deaths from fatal doses of cocaine or methamphetamine frequently occur alongside hyperthermia [1, 12, 13, 19, 21, 24, 71, 97, 98, 100].

## Behavioral Implications

### Addiction

Addiction is characterized by compulsive drug-seeking behaviors despite adverse consequences. Psychostimulants are highly addictive due to their potent effects on the dopamine system. Within the dopaminergic circuitry, the mesocorticolimbic pathway plays a key role in reward and learning related to addiction [130–133]. Specifically, the nucleus accumbens projects to the hippocampus and amygdala to facilitate the imprinting of memories and emotions. The frontal cortex acts as a decision hub, weighing information from other limbic structures to determine a course of action [59]. Other substances of abuse, such as nicotine, alcohol, opioids, and cannabinoids, target non-dopaminergic receptors to increase dopamine levels indirectly. Therefore, the mesocorticolimbic pathway and its structures are an important part of addiction research [134]. However, it is important to note that although this review is focused on dopaminergic systems, we recognize the contributions of other systems such as glutamatergic and GABAergic systems to behavioral and cognitive effects. For example, the importance of glutamate-dependent plasticity and the impact of glutamate and GABA on dopamine levels [22, 22, 41, 43], as well as stress systems in compulsion, relapse, and executive dysfunction [125, 135], have been recognized.

Animals showing signs of addiction exhibit heightened drug-seeking behaviors, risky decision-making, and disruptions to learning [136–138]. One possible explanation for the development of addiction is neuroadaptations that shift the balance between activity in the frontal cortex and other limbic structures. For example, in limbic structures, including the hypothalamus, amygdala, and VTA, methamphetamine self-administration increases levels of FosB and ΔFosB. Levels of Fos are especially increased in the hypothalamus of rats demonstrating drug-seeking behaviors [139]. Activating D3 dopamine receptors within the CA1 pyramidal neurons reduces GABA_A_ receptor-mediated transmission, resulting in less inhibitory signaling within the hypothalamus [135]. Rats with long access to cocaine self-administration have less GABA-mediated signaling and more AMPA-mediated signaling during periods of abstinence in the weeks following cocaine exposure [125]. Increased D1 dopamine receptor activity, achieved through the administration of endogenous dopamine or the agonist SKF38393, enhances long-term potentiation within the CA1 region of the hippocampus [140]. Additionally, cocaine exposure enhances signaling from the ventral hippocampus to the NAc shell [141]. In meth and cocaine users, levels of D2 receptors are decreased in the orbitofrontal cortex [82].

While the cause of addiction is not yet known, animal models of cocaine and methamphetamine self-administration serve as valuable tools to learn more about addiction [142, 143]. Ferland and Winstanley (2017) demonstrated that drug-naïve rats with a preference for risky decision-making show a greater willingness to take risks after cocaine self-administration. Moreover, this effect persisted for nearly a month after cocaine use, along with increased drug-seeking behavior [137]. In a cocaine self-administration paradigm, rats that exhibited higher locomotor activity also acquired cocaine more quickly and showed greater inhibition of DATs after self-administering cocaine [144]. In methamphetamine models, blocking the action of VMAT2 decreases methamphetamine and cue-related reinstatement of active lever pressing [145]. Inhibiting D1 receptors in the dorsal striatum by administering SCH23390 prior to methamphetamine self-administration reduces addiction-like behavior [136]. Furthermore, cocaine and methamphetamine self-administration and related drug-seeking behaviors can be inhibited by blocking D1, D2, or D3 receptors [89, 146, 147]. Aside from targeting specific proteins, reducing reactive oxygen species with scavengers significantly decreases methamphetamine-induced locomotor activity, self-administration, and methamphetamine-enhanced dopamine release in the nucleus accumbens [130]. Similarly, changes in immune response require further attention, as microglial activation increases in the striatum of mice after cocaine self-administration [148].

### Tolerance and Dependence

Tolerance to a drug is a form of plasticity where higher doses of drugs such as cocaine or methamphetamine are needed to achieve the same effect [149, 150]. This adaptation helps maintain homeostasis in the affected brain system, particularly within the dopaminergic system. Interestingly, tolerance seems to be drug-specific, as cocaine exposure does not blunt the effects of methamphetamine challenge [149]. In humans, cocaine tolerance can develop within a few hours, resulting in less subjective feelings of being high despite constant plasma cocaine levels [151–153]. Signs of abstinence can be observed for about a week after the last drug exposure, and after an equal period of abstinence, signs of tolerance wane in mouse and rat models (154, 155). However, it is not clear exactly how long tolerance lasts.

While many studies have identified potential targets for tolerance, the underlying mechanism of action requires further investigation. Researchers believed that increased body temperature influenced tolerance to methamphetamine, since hyperthermia is positively correlated with the severity of methamphetamine-induced neurotoxicity. However, several studies have demonstrated that tolerance occurs independently of hyperthermia [149, 154, 156]. Instead, DATs are alternative targets to improve our understanding of tolerance to both cocaine and methamphetamine.

In rat models, cocaine’s ability to inhibit DATs diminishes after several days of self-administration. After a brief period of abstinence, differences in DAT activity are no longer evident. However, upon re-exposure to cocaine, rats that had previously developed tolerance still show reduced DAT inhibition, even after a long abstinence period [143, 144]. In a recent study, DAT levels dropped significantly in the striatum after repeated binge doses of methamphetamine in mice. Additionally, tyrosine hydroxylase and VMAT2 levels also decreased but recovered more than DATs did [157]. Diminished dopaminergic responses in the limbic system may also result from decreased D1 receptor activity and dopamine metabolism [158]. Overall, tolerance appears to lower DAT and VMAT2 activity, seemingly reducing dopamine content to help induce tolerance [142]. Interestingly, a decrease in microglial response is associated with these protein changes and might help explain the development of tolerance [132, 156]. Alterations in neuroimmune signaling have previously been overlooked but have recently gained more attention and demand for further investigation [159]. Cocaine’s reduced ability to elicit potent dopaminergic responses results in increased cocaine intake to achieve previous effects at lower doses. Chronic cocaine use thus transitions from addiction to dependence, sharing common traits such as compulsive drug-seeking and impulsive behavior [133, 143]. Other biological changes within the mesocorticolimbic pathway after drug exposure, such as increased brain-derived neurotropic factor-mediated signaling, contribute to withdrawal symptoms and drug-seeking [160, 161]. Social, behavioral, and pharmacological disturbances, as mentioned above, aid in the diagnosis of substance use [162–164]. However, a person is not considered to have developed dependence until their quality of life suffers due to substance use, and they exhibit signs of withdrawal and tolerance [163]. Individuals dependent on psychostimulants such as methamphetamine experience psychological and behavioral issues and drug cravings, but these symptoms typically resolve after several weeks of abstinence. Additionally, cognitive function improves, even in individuals with a long history of use [74, 164, 165].

### Cognitive and Emotional Deficits

Due to their effects on the dopamine system, chronic cocaine or methamphetamine use can cause various cognitive and emotional deficits [166]. Individuals with drug dependence usually score lower on cognitive tests than healthy controls [167]. Cognitive deficits include impaired attention and focus [168, 169], memory issues [166, 170], reduced executive functioning [170–174], and diminished learning ability [168, 175]. Additionally, individuals addicted to cocaine and methamphetamine demonstrate impaired decision-making skills [166, 176]. Disruptions to cognitive function are influenced by the type of drug used, the duration of drug use, and the route of administration. Cognitive impairments are significantly worsened by polysubstance use [173, 177–179]. van der Plas et al. (2009) found that female cocaine and methamphetamine addicts had worse decision-making than males [176]. Cognitive function tends to improve the longer a person abstains from drug use [173, 180]. Treating substance use disorders with pharmacotherapies combined with behavioral interventions aimed at addressing substance-related cognitive and emotional deficits can improve clinical outcomes [181]. For example, cognitive behavioral therapy (CBT) can help drug users enhance impulse control, memory, and decision-making [173, 182]. Contingency management rewards drug users for objective signs of abstinence, reducing the reward value of drugs and instead emphasizing prizes given after a clean urine test, for instance [183]. Studies have also indicated that high-intensity interval training helps reduce drug use and associated cognitive impairments. Although several theories exist about how high-intensity interval training provides these benefits, further research is needed [184].

Emotional deficits include increased anxiety and paranoia [177, 185–187], mood disorders [186], agitation and irritability [178, 188], and emotional blunting and disrupted emotional regulation [178]. In a recent study of illicit drug users, nearly 50% of participants who had attempted suicide in the past year were methamphetamine users [189]. These emotional deficits impact daily life and productivity, affecting overall health and social relationships. Treating these deficits would improve the quality of life for drug users and also increase the likelihood of treatment success [187]. Emotional deficits are more common at higher doses, especially with positive psychotic symptoms, during longer periods of drug use, and are influenced by the route of administration [177, 178]. Similar to cognitive deficits, treatments targeting substance-related emotional deficits, along with pharmacotherapies, have been shown to be promising and effective [181]. In CBT, individuals can learn positive coping mechanisms to manage stress, cravings, and to distinguish between reality and substance-induced psychotic symptoms [182]. Contingency management combined with positive affect also promotes behavioral change by improving emotional health, leading to reduced drug use and craving [183]. Having a strong support network helps alleviate feelings of social deficits and increase intervention success [182]. Additionally, cravings, cognitive, and emotional deficits tend to diminish the longer abstinence is maintained [180]. Additional effects of cocaine and methamphetamine are shown in Table 1.

**Table 1:** Summary of key findings on the effects of cocaine and methamphetamine on TH, DAT, VMAT2, MAO, COMT proteins, and neurodegeneration in different brain regions

Substance	Effect on TH, DAT, VMAT2, MAO, and COMT	Brain regions impacted	Key findings	Reference
Cocaine	Increases dopamine transporter (DAT) mRNA	Frontal cortex	Excess synaptic dopamine leads to increased DAT upregulation and noticeable “crash”	[92]
Cocaine	Chronic exposure to cocaine decreases tyrosine hydroxylase mRNA levels and immunoreactivity	SNc, VTA NAc	Decreased mRNA levels reflect lower levels of tyrosine hydroxylase protein levels observed in chronic cocaine users	[92, 93]
Cocaine	Increased tyrosine hydroxylase activity after chronic cocaine use	VTA NAc	Due to decreased tyrosine hydroxylase protein levels, existing tyrosine hydroxylase proteins increase activity to maintain dopaminergic homeostasis	[22, 41]
Cocaine	Sensitization from repeated use exacerbates changes in DAT mRNA	VTA	Cocaine challenge after a long period of abstinence from cocaine causes significant and rapid increases in DAT mRNA or decreases depending on pretreatment	[92]
Cocaine	Differences in genotype affect dopamine-degrading enzymes and executive function	Medial prefrontal cortex (mPFC)	Pathway influences cocaine-seeking behavior and locomotor sensitization	[117]
Cocaine	Cocaine does not directly interact with VMAT2 but induces VMAT2 trafficking from the plasma membrane to the intracellular space. Reserve vesicles and vesicles near the plasma membrane are depleted	VTA, NAc	Reduced VMAT2 proteins near the plasma membrane decrease sequestration of dopamine into vesicles. Depletion of vesicular dopamine stores causes synaptic dopamine concentrations to increase	[59, 108–113]
Cocaine	Cocaine binds to dopamine transporters and inhibits their action		Extra-synaptic dopamine levels increase along with abnormal post-synaptic signaling	[53, 54]
Cocaine	Levels of synaptic dopamine transporters are increased soon after cocaine exposure. Gender differences have been noted in DAT changes	Striatum	DAT numbers are upregulated. DATs are trafficked from inside the cell to the plasma membrane, and an increase in DAT mRNA is observed to account for excess synaptic dopamine	[54, 62, 92]
Cocaine	DAT inhibition dysregulates downstream signaling cascades, leading to behavioral changes	VTA, frontal association cortex, medial prefrontal cortex	Altered signaling in the ventral tegmental area-medial prefrontal cortex-frontal association cortex pathway promotes drug-seeking behaviors and cocaine-induced locomotor sensitization	[115]
Cocaine	MAO-B is believed to play a larger role in humans for dopamine degradation compared to MAO-A	Amygdala, hippocampus, striatum, nucleus accumbens, frontal cortex	Inhibition of MAO-B reduces euphoric effects, self-administration, and dopamine metabolites	[119, 123]
Methamphetamine	Increased number of tyrosine hydroxylase-positive axons	Frontal cortex	Increased dopamine levels immediately after acute drug exposure	[71]
Methamphetamine	A reduction in neurons/terminal boutons that stain positive for tyrosine hydroxylase	VTA, NAc, frontal cortex, caudate nucleus	Chronic methamphetamine use induces losses in tyrosine hydroxylase	[93, 94]
Methamphetamine	Tyrosine hydroxylase mRNA and protein levels decrease	SNc, VTA, NAc	Repeated methamphetamine treatment reduces tyrosine hydroxylase mRNA and protein	[95, 96]
Methamphetamine	Increased tyrosine hydroxylase mRNA and protein after abstinence	VTA, SNc	Abstinence from methamphetamine after chronic use helps recuperate tyrosine hydroxylase function	[95]
Methamphetamine	A reduction in tyrosine hydroxylase expression, phosphorylation, and activity in the substantia nigra and striatum. Reduced dopamine levels in the striatum	Striatum, SNc	A reduction in dopamine levels and tyrosine hydroxylase function is observed several days after acute methamphetamine	[97]
Methamphetamine	Striatal levels of dopamine, dopamine metabolites, and tyrosine hydroxylase decrease after binge dosing in male mice	Striatum	Low dopamine levels, in addition to decreased tyrosine hydroxylase activity and dopamine metabolites, suggest that dopamine binge dosing blunts dopamine production	[98–100]
Methamphetamine	Tyrosine hydroxylase protein and phosphorylation levels are unaffected, but behavior changes and signs of anxiety are observed after a single, low methamphetamine dose in mice	Hippoc	The hippocampus is not involved in behavioral changes at low doses of meth, and protein levels are not affected, suggesting other brain structures may play a bigger role in responding to low meth doses	[101]
Methamphetamine	Higher levels of dopamine metabolites, tyrosine hydroxylase phosphorylation, and extracellular dopamine are caused by overactive tyrosine hydroxylase. Sensitivity to methamphethamine.is increased in mice	Striatum	Overproduction of dopamine and subsequent products increases sensitivity to meth-induced effects. Likely due to increased levels of dopamine available for signaling and degradation	[5, 102]
Methamphetamine	VMAT2 binds methamphetamine, reducing the amount of dopamine binding to VMAT2	Ventral tegmental area, substantia nigra, striatum	Less dopamine is stored in vesicles, reducing dopamine reserves and increasing cytosolic dopamine levels	[6, 27, 38, 103]
Methamphetamine	The number of vesicles and VMAT2 proteins is reduced by methamphetamine exposure. Susceptibility to changes to VMAT2 can be influenced by age and genetic makeup	Striatum, ventral tegmental area, substantia nigra,	Dopamine storage is disrupted, increasing cytosolic dopamine levels and oxidation	[6, 27, 28, 103, 104]
Methamphetamine	Methamphetamine blocks dopamine transporters and induces reverse transporting action	Substantia nigra, ventral tegmental área, nucleus accumbens, frontal cortex, hippocampus	Intracellular dopamine leaves the cell, and extrasynaptic dopamine is not transported back into the cell. Extrasynaptic levels of dopamine are increased, potentiating dopaminergic signaling	[6, 27, 38]
Methamphetamine	Methamphetamine causes prolonged decreases in DAT levels	Striatum	Dopamine clearance from the synapse is diminished, causing downstream effects in dopamine signaling and synthesis	[97–100]
Methamphetamine	Meth induced decreases in DAT protein levels are exacerbated by overactive TH	Substantia nigra, striatum	Proteins within dopaminergic systems influence one another. When tyrosine hydroxylase enzymes are overactive, meth-induced changes to DATs are more pronounced	[5, 114]
Methamphetamine	Repeated treatment with high doses of meth causes greater reductions in DATs compared to lower doses of meth	Striatum	Dose-dependent effects highlight the importance of decreasing drug intake and preventing dose escalation in drug users to minimize neurobiological changes	[96]
Methamphetamine	Monoamine oxidase (MAO) and catechol-O-methyltransferase (COMT) enzymes degrade dopamine. Changes to enzymatic activity is often used to manipulate dopamine levels	Prefrontal cortex, nucleus accumbens, ventral tegmental area, substantia nigra,	Both MAO and COMT are responsible for degrading excess dopamine within neurons	[37, 117–121]
Methamphetamine	MAO mediates methamphetamine induced increases in mitochondrial oxidative stress within the SNc and VTA. However, the SNc appears to be more susceptible to dopaminergic degeneration than the VTA	SNc, VTA	MAO potentiates mitochondrial oxidative stress after meth exposure. Degeneration of dopamine neurons in the SNc has implications for locomotor activity. The VTA is impacted to a lesser extent	[37, 118]
Methamphetamine	MAO-B’s role in mitochondrial oxidative stress makes it a prime target to attenuate meth-induced oxidative stress. Inhibiting voltage-gated calcium channels is also effective at mitigating mitochondrial oxidative stress	Substantia nigra, ventral tegmental area	Inhibiting the action of MAO-B or reducing Cav1 channel activity prevents SNc dopaminergic neuron degeneration	[37, 118]
Methamphetamine	COMT’s ability to degrade dopamine influences sensitivity to methamphetamine and cognitive performance. Differences in the function of degradative enzymes can occur due to changes in genetic makeup	Prefrontal cortex,	Genetic differences contribute to differential susceptibility to drug effects and dependence due to differences in dopamine degradation, dopamine levels, and metabolites. These differences also affect executive function	[117, 121, 124]

### Integration and Translational Implications

**Fig. 2:**
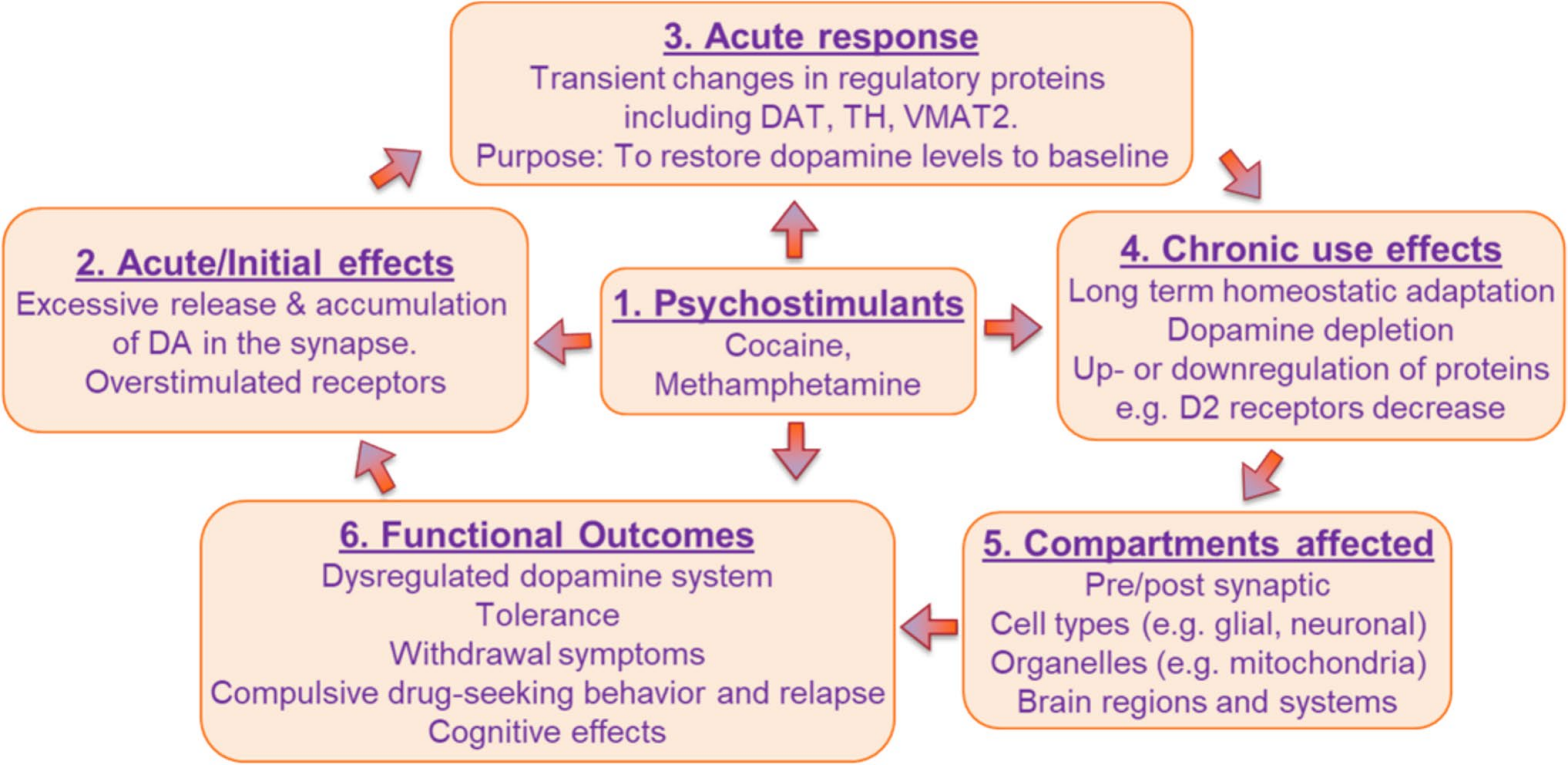
Disruption of dopamine homeostasis by psychostimulants. (1) The psychostimulants cocaine and methamphetamine disrupt dopamine homeostasis. (2) Excessive release and accumulation of dopamine occur in the synapse. (3) These acute disruptions cause transient changes in proteins such as dopamine transporters, tyrosine hydroxylase, and VMAT2, helping the system return to baseline. (4) As a result, long-term homeostatic adaptations ultimately lead to a dysregulated dopamine system. Proteins experience long-term up- or downregulation. (5) Psychostimulants have effects across many compartments, such as pre- and post-synaptic neurons, cell types, organelle types, brain regions, and systems. (6) Functional outcomes of chronic psychostimulant use include tolerance, withdrawal effects, compulsive drug-seeking behavior, and relapse. Abbreviations: DA, dopamine; DAT, dopamine transporter; TH, tyrosine hydroxylase; VMAT2, vesicular monoamine transporter 2; D2, D2-type dopamine receptors

The way a psychostimulant disrupts the dopamine system and its homeostatic regulation depends on several factors. For instance, acute exposure to psychostimulants causes temporary changes in the dopaminergic system, while long-term effects result from chronic exposure, as shown by the integrative model in Fig. 2. Different compartments, such as presynaptic neurons, post-synaptic neurons, and glia, are affected in different ways. Similarly, functional outcomes like tolerance, relapse, or changes in cognition are also variably influenced by homeostatic disruptions caused by psychostimulant exposure.

The interaction of dopamine homeostatic remodeling and neural plasticity plays an important role in addiction and affects relapse risk, as shown in Fig. 2. These factors pose translational challenges in research and outcome interpretation. As previously mentioned, and illustrated in Fig. 2, the brain adjusts homeostatically in response to drug exposure, requiring higher doses to produce the same effects—this underpins the concept of tolerance. This plasticity, where neural circuits can change, can also increase the likelihood of relapse even after long-term abstinence [143, 144]. For example, earlier research reveals that altered dopamine signaling and receptor sensitivity contribute to drug-seeking behaviors, complicating treatment efforts [89, 133, 143, 147]. Moreover, the limitations of animal models in translating findings to human addiction underscore the need for caution, since results may not always directly apply to humans. Tackling these cross-cutting issues is essential for developing effective strategies to lower relapse rates and support recovery.

## Therapeutic Interventions and Future Directions

Pharmacological interventions for psychostimulant use of cocaine and methamphetamine have been challenging [190]. Although there are currently no FDA-approved drugs to treat abuse of these psychostimulants, research is ongoing to identify and try out new medications [191]. Current therapeutic interventions are based on behavioral therapies such as cognitive-behavioral therapy (CBT), motivational interviewing, contingency management, and 12-step programs [192, 193]. These programs are effective behavioral treatments that help individuals develop coping strategies for resisting drug-induced cravings, abstain from drug use, and provide peer support and community.

Pharmacological interventions aimed at treating cocaine addiction have included disulfiram, modafinil, and topiramate [190]. These treatments are often evaluated using end points such as test subjects achieving abstinence, the duration of subsequent abstinence, as well as reductions in the frequency or quantity of cocaine used, and treatment compliance [194]. Topiramate, an anticonvulsant, is being investigated as a potential treatment to decrease cocaine craving and consumption [195–198]. However, topiramate is not a first-line treatment, as previous studies have shown no significant difference in cocaine craving or reduction of cravings between placebo and topiramate treatment, suggesting that topiramate’s effects are context-dependent and may only work for specific subgroups. It is also not well tolerated. In many cases, patients must be titrated to a desired topiramate dose while avoiding higher doses to avoid adverse effects [199–201]. Additionally, cognitive impairments in cocaine-dependent individuals may be worsened by topiramate treatment [202]. Similarly, studies on drugs such as disulfiram and modafinil have yielded mixed evidence. Disulfiram has been used in alcohol dependence and has been associated with an increased period of abstinence from concomitant cocaine and alcohol use when combined with cognitive behavioral therapy [194, 203–205]. However, disulfiram has also failed to reduce cocaine use when administered alone or combined with cognitive behavioral therapy and contingency management [194, 206]. Modafinil used to treat narcolepsy is being studied for its ability to reduce cocaine use by enhancing dopamine function [207–211]. Nonetheless, its effectiveness in reducing cocaine use and helping individuals achieve abstinence may be influenced by confounding variables, such as gender differences, resulting in performance outcomes similar to placebo treatment [212]. Topiramate, disulfiram, and modafinil’s clinical relevance as treatments for cocaine dependence appears to be context-dependent, depending on factors like whether individuals are also receiving other pharmacotherapies or behavioral therapy, and whether they use any additional drugs concurrently [213]. Current literature suggests that treatments like cognitive behavioral therapy are helpful in maintaining abstinence from cocaine use beyond any benefit conferred by the aforementioned pharmacotherapies [194, 201, 212]. Before any of these pharmacotherapies can be considered front-line treatments, they must be studied further.

For methamphetamine use disorder, promising drugs such as bupropion and naltrexone are being investigated. Bupropion is an antidepressant that affects dopaminergic signaling and has shown potential in reducing methamphetamine use [214–216]. Naltrexone, currently used to treat opioid and alcohol dependence, is also being explored for its possible benefits in treating methamphetamine use disorder [216–219]. Research is also ongoing to develop a vaccine that could generate an immune response against methamphetamine, thereby decreasing its effects on the host. One example is Entolimod, which targets toll-like receptor-5 to increase antibody production and address the behavioral and physiological effects of drugs of abuse [220]. Other immunotherapies work by reducing cytokine production to lessen neuroinflammation and addiction [221]. Ibudilast and ceftriaxone are promising immunotherapies for both cocaine and methamphetamine use disorder. They decrease drug self-administration and relapse, but further testing in humans is needed [222].

Novel pharmacological targets for PSUD are necessary. Research continues to discover new drug targets, such as allosteric modulators of dopamine receptors and compounds that impact not only the dopamine system but also other neurotransmitter systems, including glutamate or GABA [191]. For example, glutamate transporter-1 is a key target for substance use and neurological disorders and is affected by drugs such as ceftriaxone [222, 223]. Another promising research area focuses on harnessing the neuroprotective effects of novel compounds such as antioxidants that reduce oxidative stress [130]. These antioxidants can protect neurons from the neurotoxic effects of psychostimulants.

Future research should focus on understanding mechanisms, personalized medicine, and preventative strategies. The mechanisms by which psychostimulants influence the brain and behavior are complex, and much remains to be explored. Further research is needed to comprehend these mechanisms fully. Developing individualized treatment plans that take into account genetic, psychological, and environmental factors holds promise for more effective interventions. Additionally, exploring preventative measures such as early intervention and education programs remains valuable in reducing the incidence of psychostimulant abuse.

## Limitations

While this review provides valuable insights, it has several limitations. First, it mainly focuses on cocaine and methamphetamine, which may cause it to overlook the effects of other psychostimulants on dopamine homeostasis. Although the review highlights dopamine homeostasis, it also discusses the contributions of other systems, such as glutamatergic and GABAergic systems, but to a lesser extent. Due to the neurobiological complexity of the cognitive and behavioral effects of substance use, the discussion might oversimplify dopamine dysregulation and not fully capture the intricate interactions among neurotransmitters and the roles of other systems in addiction and cognitive deficits.

## Conclusion

Psychostimulants interfere with the dopamine system through mechanisms that alter dopamine synthesis, storage, release, reuptake, and degradation. These changes affect dopamine homeostasis both acutely and chronically (Fig. 2), including receptor downregulation and shifts in the expression of key transporter proteins. These alterations are associated with neurobiological effects such as receptor desensitization, changes in synaptic plasticity, oxidative stress, and potentially neuronal cell death. Disruption of dopamine homeostasis contributes to the development of tolerance, dependence, and addiction. Psychologically and behaviorally, these disruptions can cause cognitive deficits and emotional instability. However, focusing solely on dopamine balance disruptions is not enough to treat substance use disorders and their symptoms. A comprehensive approach should also target deficits caused by other monoamines or systems, including glutamatergic, GABAergic, and opioid systems.

Addressing the complex issues of psychostimulant abuse requires a multidisciplinary approach that integrates pharmacology, behavioral, and social interventions. Future research would benefit the field by clarifying mechanisms by which psychostimulants alter dopaminergic activity and CNS function, investigating neuroprotective strategies that address neurotoxicity and subsequent dopaminergic neuron death, and developing preventive strategies to reduce the prevalence of psychostimulant abuse.

## Data Availability

Not applicable.
